# Tricuspid valve leaflet multiplicity and Doppler-derived right-heart hemodynamic trajectories after surgical repair

**DOI:** 10.1016/j.xjon.2026.102089

**Published:** 2026-09-01

**Authors:** Tomohiro Iwakura, Mitsunobu Kitamura, Yoshinori Nakahara, Kanako Kishiki, Yuki Izumi, Makoto Ono, Atsushi Yano, Kohei Sumi, Akira Marui, Thilo Noack, Itaru Takamisawa, Mamoru Nanasato, Jun Shimizu, Mitsuaki Isobe

**Affiliations:** a Department of Cardiovascular Surgery, Sakakibara Heart Institute, Tokyo, Japan; b Department of Cardiology, Sakakibara Heart Institute, Tokyo, Japan; c Department of Pediatric Cardiology, Sakakibara Heart Institute, Tokyo, Japan; d Department of Cardiac Surgery, Heart Center Leipzig at the University of Leipzig, Leipzig, Germany; e Department of Anesthesiology, Sakakibara Heart Institute, Tokyo, Japan; f Sakakibara Heart Institute, Tokyo, Japan

**Keywords:** tricuspid valve repair, tricuspid regurgitation, leaflet morphology, tricuspid regurgitation pressure gradient, right ventricular function, Doppler echocardiography

## Abstract

**Objective:**

To determine whether tricuspid leaflet multiplicity is associated with divergent postoperative hemodynamic trajectories after surgical repair.

**Methods:**

One hundred seventeen patients undergoing tricuspid valve surgery (January 2023 to March 2025) were prospectively enrolled, with leaflet morphology classified by intraoperative visualization and serial echocardiography over 1 year. The prespecified primary end point was a Doppler-derived tricuspid regurgitation pressure gradient (TRPG) increase, defined as a 5-mm Hg-or-greater increase in TRPG from discharge to 1 year.

**Results:**

Among 100 patients with paired data, the primary end point occurred more frequently in the 4/5-leaflet group despite similar residual tricuspid regurgitation (TR) and discharge TRPG (48.5% vs 26.9%; odds ratio, 2.56; 95% CI, 1.07-6.12; *P* = .034); the age-adjusted association was borderline (aOR, 2.39; *P* = .054) and the within-group rise significant only in that cohort (*P* = .024). Severe-or-greater TR recurrence was rare (3.0% vs 0.0%; *P* = .327). Discharge tricuspid annular plane systolic excursion was lower in the 4/5-leaflet group (0.96 ± 0.36 vs 1.15 ± 0.36 cm; *P* = .010) but converged by 1 year (*P* = .585).

**Conclusions:**

Tricuspid leaflet multiplicity was associated with a subphenotype showing preserved early TR reduction, lower discharge tricuspid annular plane systolic excursion, and a greater 1-year frequency of Doppler-derived TRPG increase. Morphology-based stratification may refine postoperative surveillance pending external validation.

**Figure:**
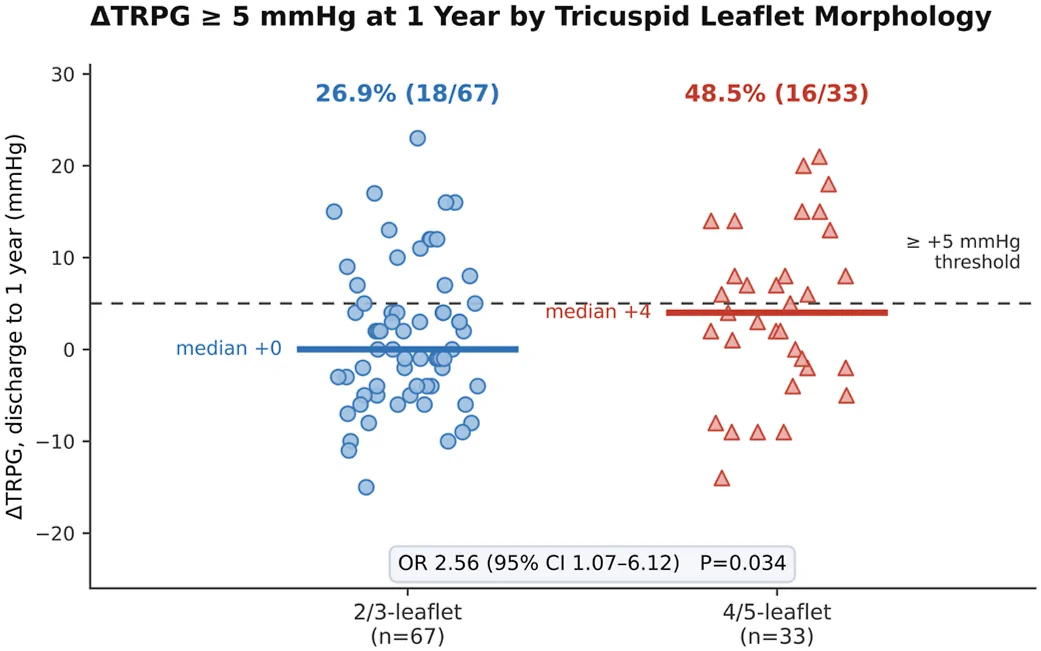
Doppler-derived TRPG increase (ΔTRPG ≥5 mm Hg) at 1 year by tricuspid leaflet morphology.

**Central Message**

Tricuspid leaflet multiplicity was associated with preserved early TR reduction, lower discharge TAPSE, and a greater frequency of Doppler-derived TRPG increase at 1 year.

**Perspective**

Tricuspid regurgitation recurs in 10 to 30% of patients within 1 year of surgical repair, yet predictors remain poorly defined. In this prospective cohort, 4- or 5-leaflet morphology marked a subphenotype with preserved early repair, lower discharge TAPSE, and a greater frequency of Doppler-derived TRPG increase at 1 year. Discharge TAPSE may serve as an early functional warning.

Contemporary tricuspid valve repair, particularly ring annuloplasty combined with adjunctive subvalvular techniques, achieves low rates of residual regurgitation at discharge,^1^ yet moderate-or-greater tricuspid regurgitation (TR) recurs in 10 to 30% of patients within the first postoperative year.^2^^,^^3^ Identifying patients who are vulnerable to delayed deterioration despite an initially successful repair remains unresolved. Reported correlates of late recurrence include leaflet tethering, persistent pulmonary hypertension, and suture-based techniques,^4^ whereas the prognostic relevance of intrinsic valve morphology has received less attention. More than 40% of human hearts harbor 4 or more functional leaflets, reflecting duplication of the anterior, posterior, or septal scallops,^5^ and in transcatheter edge-to-edge repair leaflet complexity has been associated with residual TR.^6^, ^7^, ^8^ Whether leaflet multiplicity affects postoperative right-heart recovery after surgical repair remains unknown.

We hypothesized that leaflet multiplicity identifies a vulnerable right-heart subphenotype after an apparently successful repair: patients with 4- or 5-leaflet morphology may achieve comparable early TR reduction yet subsequently develop a Doppler-derived increase in tricuspid regurgitation pressure gradient (TRPG) within 1 year.

## Methods

### Study Design and Population

This prospective, single-center observational cohort enrolled patients undergoing tricuspid valve surgery at Sakakibara Heart Institute between January 2023 and March 2025. Exclusions were emergency surgery, previous tricuspid intervention, and congenital heart disease other than atrial septal defect or patent foramen ovale. The institutional review board approved the protocol (no. 22-041, November 25, 2022); informed consent was waived using an opt-out approach, and the investigation conformed to the Declaration of Helsinki. A total of 117 patients were analyzed (114 repairs, 3 replacements).

### Intraoperative Morphology Assessment

Tricuspid valve anatomy was inspected under direct surgical visualization after completion of any concomitant left-sided procedures. The operating surgeon recorded the number of distinct leaflets, papillary muscles, chordal attachments, and commissural leaflets, with real-time independent verification by a heart-team cardiologist; discordant classifications were resolved by consensus. Two- and three-leaflet anatomies formed the 2/3-leaflet group and 4- and 5-or-more-leaflet anatomies the 4/5-leaflet group. A formal blinded interobserver reproducibility study was not undertaken. The analysis was prespecified on this binary dichotomy, which is robust to the least reproducible boundary in the framework—that between a duplicated anterior scallop and an anterior papillary muscle complex adjoining a duplicated posterior scallop—because both variants fall within the 4/5-leaflet group; subtype labels were descriptive and not used in the primary analysis.

### Surgical Repair and Echocardiographic Assessment

Ring prostheses were selected at surgeon discretion from a rigid ring (Carpentier-Edwards Physio Tricuspid; Edwards Lifesciences), a semirigid partial band (Tri-Ad 2.0; Medtronic), or a flexible complete band (SimuPlus; Medtronic); adjunctive leaflet-level and subvalvular procedures, including spiral suspension,^9^ were added on intraoperative findings, and operations used median sternotomy or right intercostal minithoracotomy. Transthoracic echocardiography was performed preoperatively, at discharge, and at 1 year per ASE recommendations^10^^,^^11^ and centrally reanalyzed by a single cardiologist blinded to morphology and outcomes. TR severity was graded on the American Society of Echocardiography/European Association of Cardiovascular Imaging integrated multiparametric scheme,^10^ in which severe-or-greater TR denotes severe, massive, or torrential regurgitation; TRPG was derived from peak TR velocity using the simplified Bernoulli equation and RV function by tricuspid annular plane systolic excursion (TAPSE).^11^ Preoperative transesophageal echocardiography assessed morphology with multiplane 2- and 3-dimensional views.^5^

### End Points and Statistical Analysis

The primary end point was a Doppler-derived TRPG increase from discharge to 1 year, defined as ΔTRPG ≥5 mm Hg. The 5-mm Hg cutoff matched the upper bound of typical Doppler TRPG test–retest variability rather than a validated minimum clinically important difference, and was not intended to define clinical pulmonary hypertension. Prespecified secondary end points were severe-or-greater TR recurrence at 1 year, continuous ΔTRPG, discharge TR and TRPG, multivariable analysis, and ring type-stratified analysis; serial TAPSE was exploratory. Of 117 patients, 100 (85.5%) had paired discharge and 1-year TRPG; 17 were excluded (11 without 1-year echocardiography, 4 with nonmeasurable TRPG owing to absent or trivial TR, and 2 missing both time points). Groups were compared using the Welch *t* test, the Mann-Whitney *U* test, or Fisher exact test as appropriate, and within-group change by the Wilcoxon signed-rank test. Logistic regression was constructed sequentially as univariable, age-adjusted, and full models with covariates selected a priori, and Firth's penalized likelihood was applied given approximately 8.5 events per variable. The primary end point was the single prespecified primary comparison and no multiplicity adjustment was applied. For analyses, we used R, version 4.3, or greater. Sensitivity analyses and full modeling details are given in Appendix E1.

## Results

### Population and Morphology

Among 117 patients (median age, 74 years; interquartile range, 68-79 years, 49% women), direct intraoperative visualization identified 3-leaflet anatomy in 72 (61.5%) and 2-leaflet anatomy in 3 (2.6%), constituting the 2/3-leaflet group (n = 75, 64.1%). The remaining 42 (35.9%) had 4/5-leaflet morphology: duplicated anterior leaflet in 24, duplicated posterior leaflet in 12, duplicated septal leaflet in 2, and 5-or-more leaflets in 4 (Figure 1).

**Figure 1:**
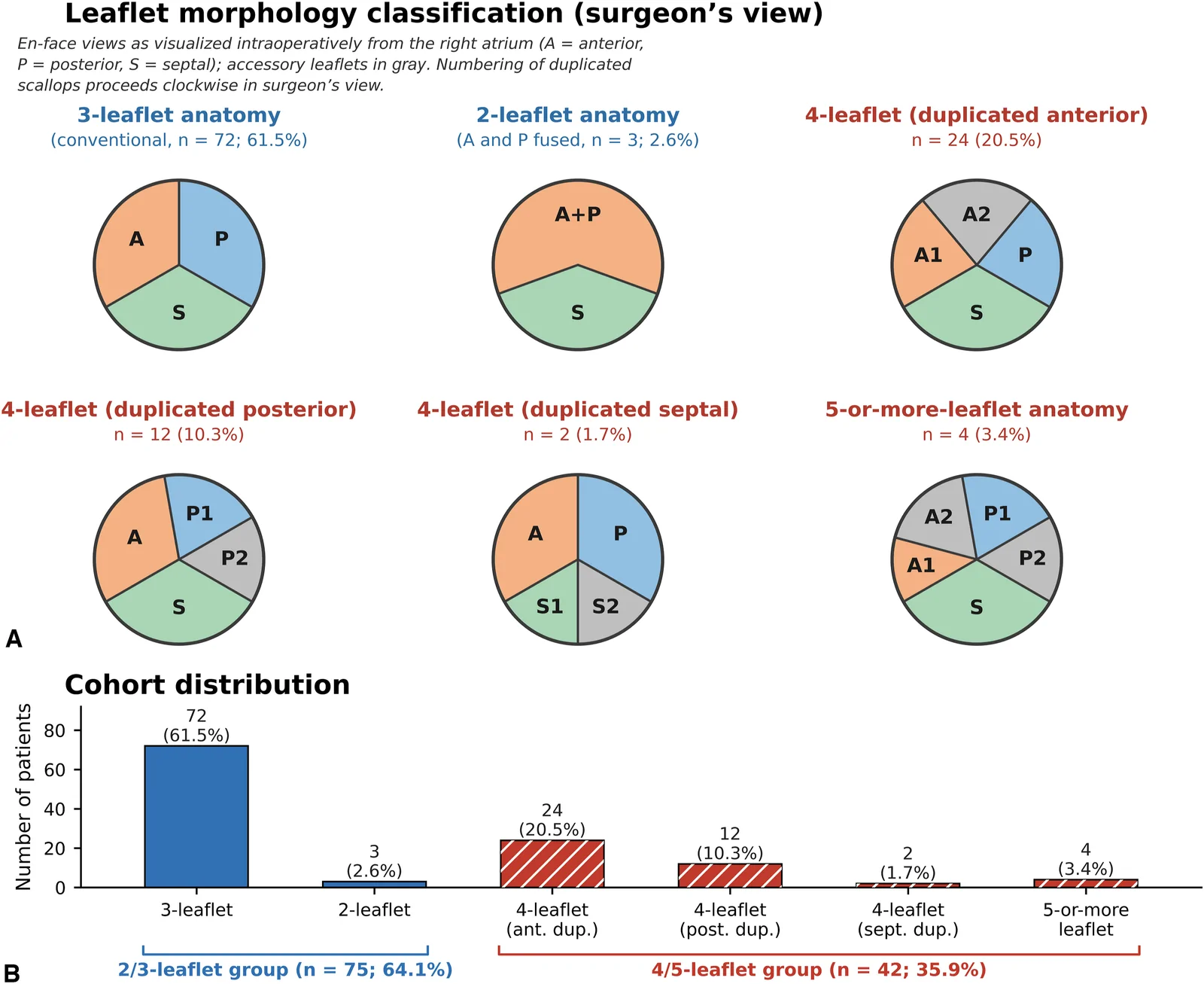
Tricuspid valve leaflet morphology classification and cohort distribution. A, En-face schematics from the surgeon's perspective with the septal leaflet at the bottom, showing 3-leaflet and 2-leaflet anatomy, 4-leaflet variants with a duplicated anterior, posterior, or septal leaflet, and 5-or-more-leaflet anatomy; accessory leaflets are *gray* and duplicated scallops numbered clockwise. B, Distribution among the 117 patients. Morphology was assigned intraoperatively in every patient, so no data are missing.

### Baseline Characteristics

Patients with 4/5-leaflet morphology were modestly older (75.1 ± 6.6 vs 70.4 ± 10.6 years, *P* = .004), of smaller body habitus (*P* = .017), and carried a greater European System for Cardiac Operative Risk Evaluation II (3.98 [1.99-5.50] vs 2.50 [1.58-4.73], *P* = .037; Table 1). Preoperative right-heart burden was nonetheless similar: TRPG (33.5 ± 13.0 vs 35.9 ± 13.6 mm Hg, *P* = .338), TAPSE (2.01 ± 0.53 vs 1.87 ± 0.55 cm, *P* = .215), annular diameter (*P* = .683), and prevalence of severe-or-greater TR (*P* = .665). Levels of serum creatinine were virtually identical (*P* = .945), whereas calculated creatinine clearance was lower in the 4/5-leaflet group (46.0 ± 17.9 vs 56.3 ± 25.5 mL/min, *P* = .011), reflecting older age and smaller body size rather than intrinsic renal disease; this, with more New York Heart Association functional class III or IV (45.2% vs 32.0%) and greater procedural weight, accounts for the greater European System for Cardiac Operative Risk Evaluation II scores.

**Table 1:** Baseline clinical and echocardiographic characteristics

Variable	2/3-leaflet (N = 75)	4/5-leaflet (N = 42)	*P* value
Age, y	70.4 ± 10.6	75.1 ± 6.6	.004
Female, n (%)	33 (44.0)	24 (57.1)	.184
Body surface area, m^2^	1.63 ± 0.21	1.54 ± 0.18	.017
Atrial fibrillation, n (%)	53 (70.7)	32 (76.2)	.666
NYHA functional class III or IV, n (%)	24 (32.0)	19 (45.2)	.167
Chronic kidney disease, n (%)	32 (42.7)	24 (57.1)	.177
Secondary TR etiology, n (%)	66 (88.0)	34 (81.0)	.601
Severe-or-greater preoperative TR, n/N (%)	20/75 (26.7)	12/39 (30.8)	.665
EuroSCORE II, %	2.50 [1.58-4.73]	3.98 [1.99-5.50]	.037
STS-PROM, %	3.8 [2.0-7.4]	4.1 [3.4-6.9]	.345
TRI-SCORE, %	3 [2-8]	4 [2-8]	.339
Preoperative TRPG, mm Hg	35.9 ± 13.6	33.5 ± 13.0	.338
Preoperative TAPSE, cm	1.87 ± 0.55	2.01 ± 0.53	.215
Preoperative RV-FAC, %	37.7 ± 6.2	38.6 ± 7.4	.492
Preoperative LVEF, %	58.9 ± 10.4	58.9 ± 8.4	.968
Tricuspid annular diameter, mm	39.5 ± 4.9	40.1 ± 7.6	.683
Serum creatinine, mg/dL	1.26 ± 1.37	1.28 ± 1.32	.945
Creatinine clearance, mL/min	56.3 ± 25.5	46.0 ± 17.9	.011

Data are mean ± SD, median [interquartile range], or n (%), compared by the Welch *t* test, Mann-Whitney *U* test, or the Fisher exact test. Severe-or-greater TR denotes grade ≥3+, corresponding to severe-or-greater regurgitation by American Society of Echocardiography/European Association of Cardiovascular Imaging criteria. EuroSCORE II was available in 91 of 117 patients and annular diameter in 70 of 117; all other variables had ≤6% missingness. The complete baseline table is provided in Table E4. Comparisons are reported throughout as 4/5-leaflet versus 2/3-leaflet morphology. *NYHA*, New York Heart Association; *TR*, tricuspid regurgitation; *EuroSCORE*, European System for Cardiac Operative Risk Evaluation; *STS-PROM*, Society of Thoracic Surgeons Predicted Risk of Mortality; *TRPG*, tricuspid regurgitation pressure gradient; *TAPSE*, tricuspid annular plane systolic excursion; *RV-FAC*, right ventricular fractional area change; *LVEF*, left ventricular ejection fraction.

### Surgical Procedures

Among 114 annuloplasty recipients, the rigid Physio ring was used in 29 of 40 (72.5%) versus 35 of 74 (47.3%) (*P* = .011), and spiral suspension was more frequent in the 4/5-leaflet group (5/42 [11.9%] vs 1/75 [1.3%], *P* = .022). The 4/5-leaflet group less often received a minimally invasive approach (9.5% vs 34.7%, *P* = .004) and more often underwent concomitant Cox-maze (23.8% vs 5.3%, *P* = .006), yet crossclamp and bypass times did not differ (Table E1). Minimally invasive cases required longer crossclamp (median 131 vs 108 minutes) and bypass (169 vs 150 minutes) times; because this approach was concentrated in the 2/3-leaflet group, its added operative burden offset the greater concomitant-procedure burden of the 4/5-leaflet group.

### Discharge Outcomes

At discharge, severe-or-greater residual TR was absent in both groups and one 4/5-leaflet patient had moderate residual TR (1/40, 2.5%). Mean discharge TRPG did not differ (19.6 ± 8.4 vs 19.2 ± 7.7 mm Hg, *P* = .79), and in-hospital mortality was 2.4% versus 1.3% (*P* = 1.00). In the prespecified exploratory analysis, discharge TAPSE was lower in the 4/5-leaflet group (0.96 ± 0.36 vs 1.15 ± 0.36 cm, *P* = .010; Figure E2), indicating greater early right ventricular impairment despite comparable TR reduction.

### Primary End Point

TRPG trajectories diverged during the first postoperative year, with the 4/5-leaflet group rising from a comparable discharge baseline (Figure 2). Among 100 patients with paired data, the primary end point occurred in 16 of 33 (48.5%) of the 4/5-leaflet versus 18 of 67 (26.9%) of the 2/3-leaflet group (odds ratio, 2.56; 95% CI, 1.07-6.12; *P* = .034). Median ΔTRPG was +4.0 mm Hg [IQR −2.0 to +8.0] in the 4/5-leaflet group (Wilcoxon *P* = .024) versus 0.0 mm Hg [−4.0 to +5.0] in the 2/3-leaflet group (*P* = .381), although the between-group continuous comparison was nonsignificant (*P* = .111). The prespecified missingness sensitivity analysis retained significance (45.7% vs 26.1%; odds ratio, 2.39; 95% CI, 1.01-5.61; *P* = .046). Compared with the 100 analyzed patients, the 17 excluded patients had a greater prevalence of chronic kidney disease (76.5% vs 43.0%; *P* = .016), a larger preoperative tricuspid annular diameter (46.2 ± 8.2 vs 38.8 ± 5.2 mm; *P* = .027, 9 evaluable), and a lower preoperative TRPG (29.5 ± 6.8 vs 36.0 ± 14.0 mm Hg; *P* = .004), the last reflecting the exclusion of 4 patients in whom TR was absent or trivial; other characteristics did not differ (Table E2).

**Figure 2:**
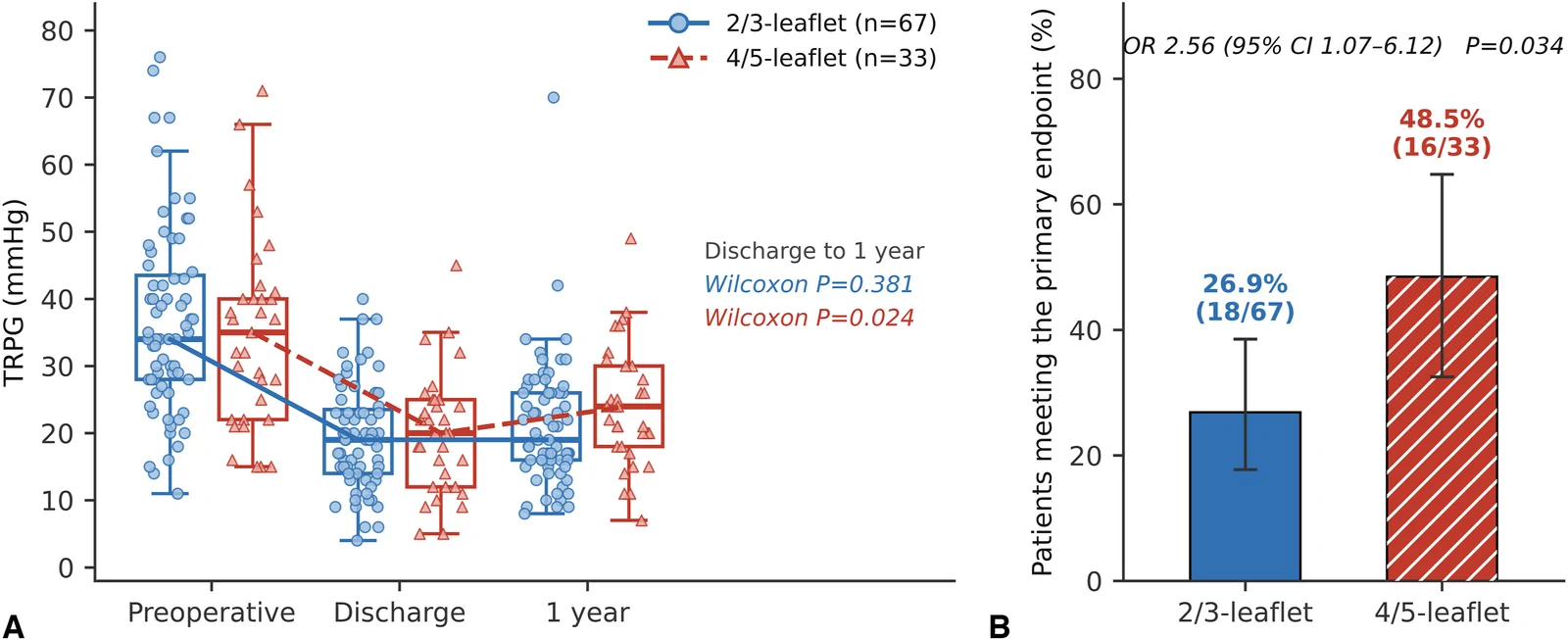
Divergent hemodynamic trajectories over the first postoperative year. A, Box-and-whisker dot plot of TRPG at the preoperative, discharge, and 1-year time points; *boxes* show the median and interquartile range, *whiskers* 1.5 times the interquartile range, with individual values overlaid. B, Proportion meeting the primary end point, a Doppler-derived tricuspid regurgitation pressure gradient increase of ≥5 mm Hg from discharge to 1 year: 48.5% (16/33) versus 26.9% (18/67) (odds ratio, 2.56; 95% CI, 1.07-6.12; *P* = .034); error bars are Wilson 95% CIs. Analyses are restricted to the 100 patients with paired discharge and 1-year measurements (4/5-leaflet n = 33, 2/3-leaflet n = 67); no imputation was performed for the 17 patients without paired data, who are compared with the analyzed cohort in Table E2. *TRPG*, Tricuspid regurgitation pressure gradient.

At 1 year, mean TRPG was 24.0 ± 9.0 versus 21.1 ± 9.3 mm Hg (*P* = .133; median 24.0 [18.0-30.0] vs 19.0 [16.0-26.0], *P* = .072), whereas TAPSE was comparable (1.31 ± 0.46 vs 1.36 ± 0.45 cm; *P* = .585) and its change from discharge to 1 year did not differ (+0.32 ± 0.54 vs +0.23 ± 0.46 cm; *P* = .411) (Table 2).

**Table 2:** One-year echocardiographic outcomes by leaflet morphology

Variable	2/3-leaflet	4/5-leaflet	*P* value
1-y TRPG, mm Hg	21.1 ± 9.3 (n = 69)	24.0 ± 9.0 (n = 33)	.133
1-y TRPG, median [IQR], mm Hg	19.0 [16.0-26.0]	24.0 [18.0-30.0]	.072
1-y TAPSE, cm	1.36 ± 0.45 (n = 64)	1.31 ± 0.46 (n = 33)	.585
ΔTRPG, discharge to 1 y, median [IQR], mm Hg	0.0 [−4.0 to 5.0] (n = 67)	4.0 [−2.0 to 8.0] (n = 33)	.111
Within-group P for ΔTRPG	.381	.024	–
ΔTAPSE, discharge to 1 y, cm	+0.23 ± 0.46 (n = 64)	+0.32 ± 0.54 (n = 32)	.411

Between-group comparisons used the Welch *t* test for means, the Mann-Whitney *U* test for medians, and the Wilcoxon signed-rank test for within-group change. Denominators differ by variable because 1-year measurements were not obtained in all patients; no imputation was performed and all analyses are complete-case. *TRPG*, Tricuspid regurgitation pressure gradient; *IQR*, interquartile range; *TAPSE*, tricuspid annular plane systolic excursion.

### Secondary End Points and Prespecified Analyses

Severe-or-greater TR at 1 year was infrequent (1/33 [3.0%] vs 0/68 [0.0%]; *P* = .327), the single recurrence occurring in a 4-leaflet patient. Leaflet multiplicity remained directionally associated with the primary end point after age adjustment (adjusted odds ratio [aOR], 2.39; 95% CI, 0.99-5.81; *P* = .054), was attenuated in the full model (aOR, 2.06; 95% CI, 0.81-5.28; *P* = .131), and rigid ring use was associated with the end point (aOR, 2.60; 95% CI, 1.02-6.61; *P* = .045) (Table 3). Firth penalized regression, threshold sensitivity analyses at 3 and 10 mm Hg, the continuous ΔTRPG comparison, and ring type-stratified estimates were all directionally consistent without reaching significance (Tables 3 and E3), and overall survival did not differ (Figure E1). One-year outcomes of the principal concomitant procedures did not differ: residual moderate-or-greater mitral regurgitation 11.1% versus 2.5% (*P* = .225) and sinus rhythm 52.8% versus 39.7% (*P* = .217), the latter consistent with the more frequent concomitant Cox-maze. Procedure-specific outcomes, in particular coronary graft patency and detailed aortic-procedure outcomes, were not systematically adjudicated.

**Table 3:** Multivariable and sensitivity analyses for the primary end point

Logistic regression (paired n = 100)	Univariable OR (95% CI), *P*	Age-adjusted aOR (95% CI), *P*	Full model aOR (95% CI), *P*
Variable			
4/5-leaflet morphology	2.56 (1.07-6.12), .034	2.39 (0.99-5.81), .054	2.06 (0.81-5.28), .131
Age (per y)	–	Included	1.01 (0.96-1.06)
Rigid Physio Tricuspid ring	–	–	2.60 (1.02-6.61), .045
Concomitant spiral suspension	–	–	0.91 (0.16-5.38), .918

Analysis	2/3-leaflet, n/N (%)	4/5-leaflet, n/N (%)	OR (95% CI), *P*
Sensitivity analyses			
Primary analysis (ΔTRPG ≥5 mm Hg)	18/67 (26.9)	16/33 (48.5)	2.56 (1.07-6.12), .034
Missingness sensitivity (n = 104)	18/69 (26.1)	16/35 (45.7)	2.39 (1.01-5.61), .046
Firth penalized regression (full model)	–	–	2.01 (0.79-5.12), .144
Threshold ΔTRPG ≥3 mm Hg	27/67 (40.3)	18/33 (54.5)	1.78 (0.77-4.12), .180
Threshold ΔTRPG ≥10 mm Hg	12/67 (17.9)	8/33 (24.2)	1.47 (0.53-4.03), .458
Continuous ΔTRPG (mean), mm Hg	1.64	4.12	Difference +2.48, .216

Covariates were prespecified on clinical and biologic plausibility rather than univariable screening; ORs are Wald-based and the model is explanatory rather than predictive. The full model showed acceptable goodness-of-fit (Hosmer-Lemeshow *P* = .85) and modest discrimination (c-statistic 0.68). The missingness sensitivity analysis reclassifies the 4 patients with non-measurable 1-year TRPG as nonevents. Stratified analysis by ring type and severe-or-greater TR recurrence are presented in Table E3. *OR*, Odds ratio; *aOR*, adjusted odds ratio; *TRPG*, tricuspid regurgitation pressure gradient; *TR*, tricuspid regurgitation.

### RV Functional Trajectory

TAPSE decreased after repair more markedly in the 4/5-leaflet group (preoperative to discharge, −1.08 ± 0.56 vs −0.73 ± 0.52 cm; *P* = .003), then recovered comparably and converged by 1 year. The primary end point therefore occurred despite substantial recovery of right ventricular function from a lower discharge baseline.

## Discussion

### Principal Findings

This prospective cohort yielded 3 principal findings. First, preoperative right-heart status and early TR reduction were comparable between morphology groups, yet discharge TAPSE was significantly lower in the 4/5-leaflet group, an early signal of RV systolic vulnerability. Second, over the subsequent year this comparability dissolved, with the primary end point occurring more frequently in the 4/5-leaflet group and a significant within-group TRPG increase only in that cohort, although continuous ΔTRPG did not differ. Third, this divergence occurred despite substantial TAPSE recovery and convergence at 1 year. These observations support a descriptive framework of latent hemodynamic vulnerability, in which a discharge functional signal precedes overt hemodynamic divergence by 1 year. To our knowledge, no previous prospective surgical cohort has examined tricuspid leaflet multiplicity in relation to postoperative Doppler-derived TRPG increase.

### Mechanistic Considerations

A central paradox is the dissociation between RV systolic function and pulmonary hemodynamics: TAPSE recovered to comparable levels by 1 year, yet TRPG increased only in the 4/5-leaflet group. This is compatible with, but does not prove, disruption of RV–pulmonary arterial coupling^12^^,^^13^; TAPSE is load- and geometry-dependent and reflects only longitudinal contraction, and formal coupling assessment would require paired afterload measurements not available here. Anatomical features of multileaflet configurations may amplify this vulnerability: increased commissural circumference reduces coaptation reserve, accessory papillary muscles introduce asymmetric chordal tension, and posterior-leaflet duplication concentrates redundant tissue where annular dilatation preferentially progresses.^14^

Whether leaflet multiplicity reduces the mechanical efficiency of the repair, marks a more vulnerable right ventricle, or reflects RV systolic recovery driving the TRPG rise cannot be resolved by these data, but 3 observations bear on the alternatives. Early repair quality was equivalent, with no severe-or-greater residual TR and comparable discharge TRPG (*P* = .79), and severe-or-greater TR recurrence remained rare and similar (*P* = .327), so the later divergence occurred without a demonstrable increase in regurgitant load. TAPSE recovery from discharge to 1 year did not differ (*P* = .41), even though TRPG increased only in the 4/5-leaflet group; if RV recovery alone determined the trajectory, comparable recovery would predict comparable TRPG increases. Conversely, the greater decrease in TAPSE from baseline to discharge in the 4/5-leaflet group (−1.08 ± 0.56 vs −0.73 ± 0.52 cm; *P* = .003), despite equivalent TR reduction, is compatible with an RV less able to tolerate acute perioperative loading changes, and experimental evidence that chronic RV pressure overload remodels the leaflet apparatus^15^ raises the possibility that both features share a common upstream pathology, including reverse causation. We therefore advance latent hemodynamic vulnerability as a descriptive framework rather than a mechanistic claim, and regard leaflet multiplicity as a clinically useful marker rather than an established cause.

### Procedural Considerations

In exploratory multivariable analysis, the rigid Physio ring use was associated with the primary end point (aOR, 2.60; *P* = .045). Ring selection was nonrandomized and preferentially applied to patients with more severe baseline TR and more complex repair phenotypes, including open sternotomy and concomitant maze, so this finding likely reflects procedural complexity rather than an independent biomechanical determinant.

### Clinical Implications

These observations raise the hypothesis that, in multileaflet morphology, postoperative assessment might extend beyond residual TR at discharge to the longitudinal TRPG trajectory, with dedicated reassessment at 6 months and 1 year. Because the prognostic link between this Doppler-derived signal and hard clinical end points was not established here, these considerations are hypothesis-generating and require prospective confirmation. Earlier detection of an increasing trajectory might permit guideline-based optimization of volume status, rhythm or rate control, and left-sided filling pressures before frank recurrence. Reliable preoperative identification of leaflet multiplicity is also needed; the difficulty of resolving accessory scallops on transthoracic echocardiography^5^ underscores the value of 3-dimensional transesophageal imaging for surgical planning, and subvalvular interventions targeting posterolateral tethering, including spiral suspension, are biomechanically rational for this substrate.^9^ The trajectory beyond 1 year could not be assessed because protocol echocardiography ended at 1 year. Either a plateau, if the increase reflects normalization of pulmonary vascular tone after relief of preoperative volume overload, or sustained elevation of afterload favoring late recoaptation failure,^4^ is plausible; a 2-time point comparison cannot establish continuing progression, and longer follow-up is required.

### Limitations

Our study had a few limitations. First, events were few, with 34 primary end-point events and 1 severe-or-greater TR recurrence, limiting the precision of multivariable estimates; full-model results, including the attenuated leaflet association and the observed rigid-ring association, should be interpreted as exploratory. Second, the 5-mm Hg threshold was an operational cutoff, not a validated minimum clinically important difference, and sensitivity analyses using alternative thresholds and continuous ΔTRPG were directionally consistent without reaching significance. TRPG is a Doppler surrogate of pulmonary arterial systolic pressure rather than a catheterization diagnosis, and the primary end point should not be interpreted as catheter-confirmed pulmonary hypertension. Third, in an exploratory analysis the primary end point was not associated with heart-failure readmission (5.9% [2/34] vs 9.1% [6/66]; *P* = .71), and no deaths occurred among patients meeting it. With only 8 readmissions and 1 death, this comparison was substantially underpowered and is presented descriptively rather than as evidence of a null effect; longitudinal New York Heart Association functional class was not systematically recorded. Moreover, because the end point is ascertained only in patients surviving to and measured at 1 year, the most severely affected patients—including 1 patient with 4/5-leaflet morphology who died of heart failure on postoperative day 109—are excluded from its denominator. Whether a 5-mm Hg Doppler TRPG increase predicts hard clinical outcomes therefore remains undetermined.

Fourth, paired data were less complete in the 4/5-leaflet group, and although the prespecified missingness sensitivity analysis was directionally consistent, differential attrition remains a potential source of bias; because the excluded patients were, if anything, more comorbid and had larger tricuspid annuli (Table E2), their omission would bias the observed association toward the null, but informative dropout cannot be excluded. Residual confounding from concomitant procedures also cannot be excluded, because event numbers were small, standardized procedure-specific outcomes were not systematically adjudicated, and no formal blinded reproducibility study of the morphology classification was performed.

Finally, the single-center design in a Japanese tertiary referral population limits external generalizability. Validation in larger, preferably multicenter cohorts with structured functional and clinical outcomes will be essential before any independent predictive, causal, or practice-changing inference can be drawn.

## Conclusions

In this prospective single-center cohort, 4- or 5-leaflet tricuspid morphology was associated with a greater proportion of patients meeting the 5-mm Hg TRPG-increase threshold at 1 year despite apparently successful early TR reduction, and lower discharge TAPSE may represent an early functional signal preceding overt hemodynamic divergence. The association persisted directionally after age adjustment and in sensitivity analyses although attenuated in adjusted and penalized models; we therefore interpret these findings as a morphology-related vulnerability signal rather than conclusive evidence of independent causation. Pending external validation, morphology-based stratification may refine postoperative surveillance.

### Declaration of Generative AI and AI-Assisted Technologies in the Writing Process

During the preparation of this work, the authors used ChatGPT (OpenAI) and Claude (Anthropic) in order to refine language, review manuscript structure, and check internal consistency. The authors did not use any generative AI or AI-assisted technologies to create, alter, or analyze scientific images, figures, graphical abstracts, or quantitative analyses. After using these tools, the authors reviewed and edited the content as needed and take full responsibility for the content of the publication.

### Webcast

You can watch a Webcast of this AATS meeting presentation by going to: https://www.aats.org/resources/intraoperative-leaflet-morphol-12240.

**Figure:**
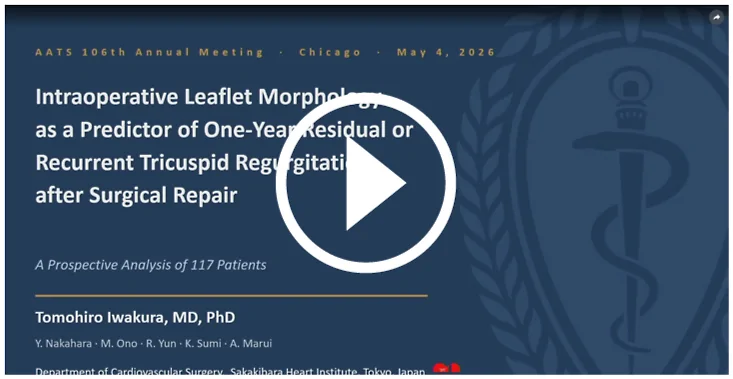

## Conflict of Interest Statement

The authors reported no conflicts of interest.

The *Journal* policy requires editors and reviewers to disclose conflicts of interest and to decline handling or reviewing manuscripts for which they may have a conflict of interest. The editors and reviewers of this article have no conflicts of interest.

## Figures and Tables

**Figure E1:**
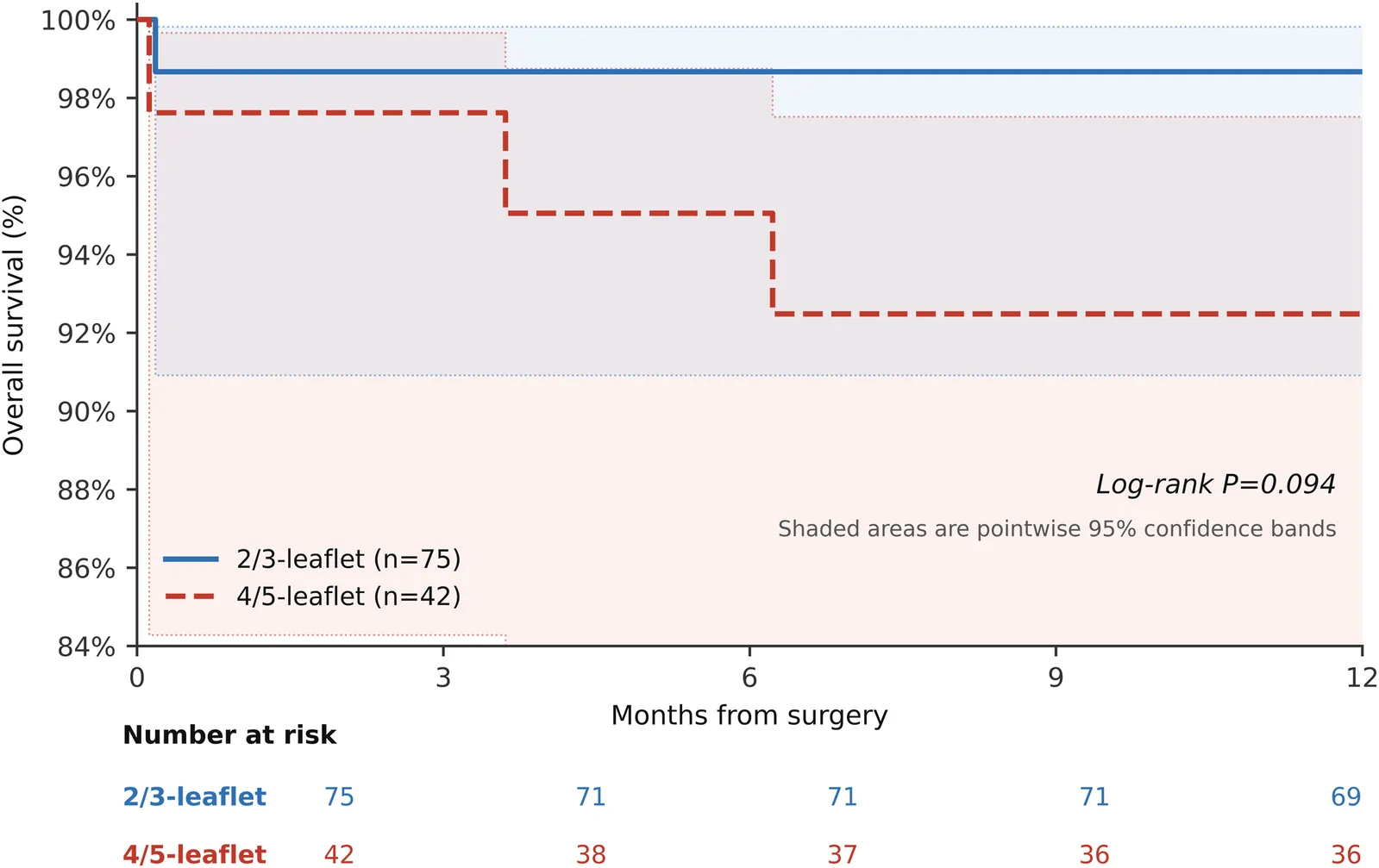
Kaplan-Meier estimates of overall survival by morphology, with numbers at risk and 95% confidence bands. All 117 patients had complete survival follow-up.

**Figure E2:**
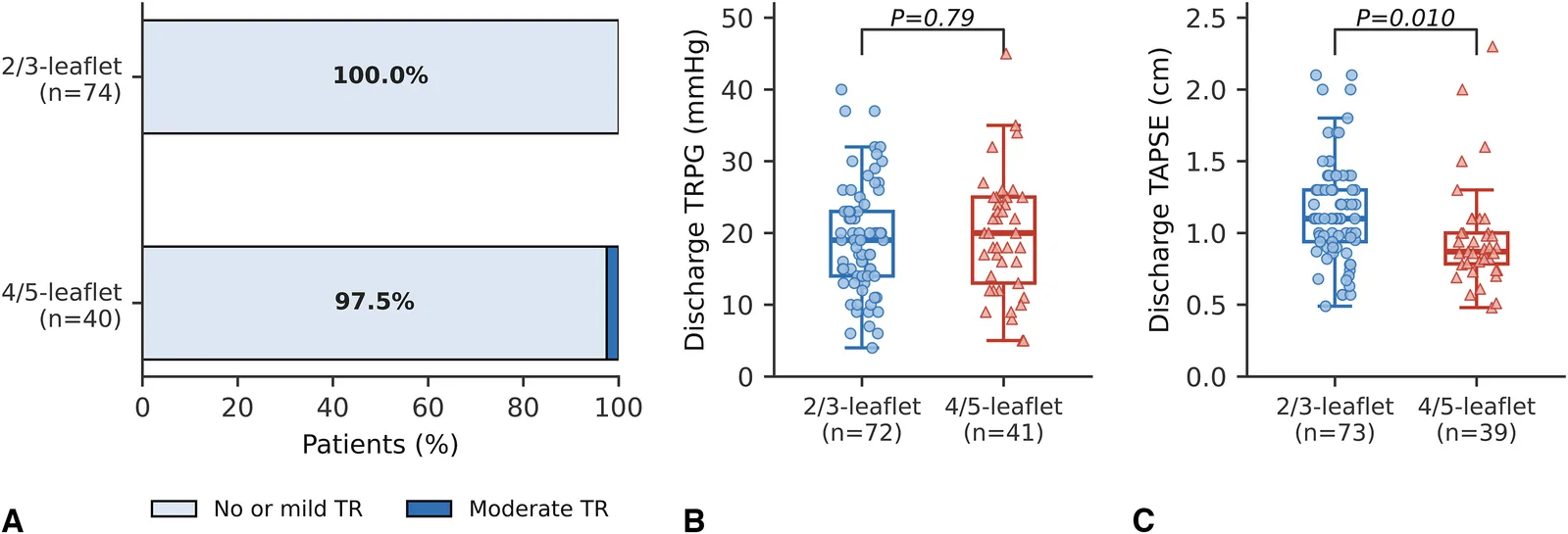
Discharge echocardiographic outcomes by morphology. A, Discharge tricuspid regurgitation (*TR*) grade. B, Box-and-whisker dot plot of discharge tricuspid regurgitation pressure gradient (TRPG; *P* = .79). C, Discharge tricuspid annular plane systolic excursion (*TAPSE*) was lower in the 4/5-leaflet group (0.96 ± 0.36 cm vs 1.15 ± 0.36 cm; *P* = .010). Denominators (4/5-leaflet vs 2/3-leaflet) were 40/74, 41/72, and 39/73 for (A-C); patients without an evaluable discharge study were excluded from that panel only, without imputation.

**Table E1:** Surgical procedures and operative outcomes

Variable	2/3-leaflet (N = 75)	4/5-leaflet (N = 42)	*P* value
Tricuspid procedure type			
Annuloplasty (with or without leaflet repair)	74 (98.7)	40 (95.2)	.292
Valve replacement	1 (1.3)	2 (4.8)	.292
Annuloplasty ring type (2/3 n = 74, 4/5 n = 40)			
Rigid Physio Tricuspid ring	35 (47.3)	29 (72.5)	.011
Flexible Tri-Ad band	38 (51.4)	11 (27.5)	.018
SimuPlus annuloplasty band	1 (1.4)	0 (0.0)	1.000
Subvalvular and leaflet-level procedures			
Spiral suspension	1 (1.3)	5 (11.9)	.022
Edge-to-edge repair	5 (6.7)	5 (11.9)	.492
Surgical approach			
Minimally invasive (right intercostal) approach	26 (34.7)	4 (9.5)	.004
Concomitant procedures			
Mitral valve repair or replacement	40 (53.3)	18 (42.9)	.336
Aortic valve replacement	19 (25.3)	17 (40.5)	.099
Coronary artery bypass grafting	11 (14.7)	8 (19.0)	.605
Cox-maze procedure	4 (5.3)	10 (23.8)	.006
Left atrial appendage closure	31 (41.3)	26 (61.9)	.036
Crossclamp time, min	111 [91-148]	116 [90-144]	.962
Cardiopulmonary bypass time, min	152 [125-198]	155 [119-195]	.856
Operation time, min	244 [185-297]	228 [181-304]	.991
In-hospital mortality	1 (1.3)	1 (2.4)	1.000

Data are n (%) or median [interquartile range], compared by the Fisher exact test or the Mann-Whitney *U* test. Annuloplasty ring type comparisons exclude the 3 patients who underwent tricuspid valve replacement (1 in the 2/3-leaflet group, 2 in the 4/5-leaflet group), so the denominators are all annuloplasty recipients. In-hospital mortality occurred in 1 patient per group (acute mesenteric ischemia in the 2/3-leaflet group; low cardiac output syndrome in the 4/5-leaflet group). Comparisons are reported as 4/5-leaflet versus 2/3-leaflet morphology.

**Table E2:** Baseline characteristics of the analyzed cohort and the excluded patients

Variable	Analyzed cohort (n = 100)	Excluded patients (n = 17)	*P* value
Age, y	71.6 ± 10.0	75.3 ± 6.5	.057
Female, n (%)	49 (49.0)	8 (47.1)	1.000
Body mass index, kg/m^2^	21.7 ± 3.1	24.8 ± 12.4	.314
Body surface area, m^2^	1.6 ± 0.2	1.7 ± 0.3	.349
Sinus rhythm, n (%)	26 (26.0)	5 (29.4)	.771
Atrial fibrillation, n (%)	74 (74.0)	11 (64.7)	.556
NYHA class III or IV, n (%)	37 (37.0)	6 (35.3)	1.000
Hypertension, n (%)	26 (26.0)	4 (23.5)	1.000
Diabetes mellitus, n (%)	14 (14.0)	3 (17.6)	.712
Chronic kidney disease, n (%)	43 (43.0)	13 (76.5)	.016
Previous stroke, n (%)	9 (9.0)	2 (11.8)	.661
Chronic obstructive pulmonary disease, n (%)	2 (2.0)	2 (11.8)	.100
Liver cirrhosis, n (%)	2 (2.0)	1 (5.9)	.378
Coronary artery disease, n (%)	14 (14.0)	4 (23.5)	.295
Secondary TR etiology, n (%)	85 (85.0)	15 (88.2)	1.000
Severe-or-greater preoperative TR, n (%)	28 (28.0)	4 (23.5)	1.000
EuroSCORE II, %	2.83 [1.69-5.22]	3.10 [2.23-4.59]	.548
STS-PROM, %	3.83 [2.30-6.98]	4.82 [3.45-5.40]	.538
TRI-SCORE, %	3.00 [2.00-8.00]	3.00 [2.00-8.00]	.736
Preoperative TRPG, mm Hg	36.0 ± 14.0	29.5 ± 6.8	.004
Preoperative TAPSE, cm	1.9 ± 0.5	2.0 ± 0.6	.827
Preoperative RV-FAC, %	38.0 ± 6.4	37.9 ± 8.1	.945
Preoperative LVEF, %	58.7 ± 10.0	59.9 ± 7.7	.569
Preoperative tricuspid annular diameter, mm	38.8 ± 5.2	46.2 ± 8.2	.027
Preoperative serum creatinine, mg/dL	1.2 ± 1.3	1.5 ± 1.5	.418
4/5-leaflet morphology, n (%)	33 (33.0)	9 (52.9)	.170
Minimally invasive approach, n (%)	26 (26.0)	4 (23.5)	1.000
Concomitant spiral suspension, n (%)	6 (6.0)	0 (0.0)	.591

Data are mean ± SD, median [interquartile range], or n (%). Continuous variables were compared by the Welch *t* test or the Mann-Whitney *U* test according to distribution; categorical variables by the Fisher exact test. The analyzed cohort comprises the 100 patients with paired discharge and 1-year TRPG measurements; the 17 excluded patients comprise 11 without 1-year echocardiography, 4 with nonmeasurable TRPG owing to absent or trivial regurgitation, and 2 missing both time points. Denominators for variables with missing observations (analyzed cohort vs excluded patients) were: EuroSCORE II 78 versus 13; STS-PROM 44 versus 9; TRI-SCORE 99 versus 17; preoperative tricuspid annular diameter 61 versus 9; preoperative TAPSE 95 versus 15; preoperative RV-FAC 99 versus 15. All other variables were complete. Three differences reached statistical significance. The higher prevalence of chronic kidney disease and the larger tricuspid annular diameter in the excluded patients indicate a more advanced disease state, so their omission would be expected to bias the observed association toward the null. The lower preoperative TRPG in the excluded patients is a mechanical consequence of the exclusion criteria, because 4 of the 17 were excluded precisely because regurgitation was absent or trivial and TRPG was therefore not measurable. The annular diameter comparison rests on 9 evaluable excluded patients and should be interpreted with corresponding caution. *NYHA*, New York Heart Association; *TR*, tricuspid regurgitation; *EuroSCORE*, European System for Cardiac Operative Risk Evaluation; *STS-PROM*, Society of Thoracic Surgeons Predicted Risk of Mortality; *TRPG*, tricuspid regurgitation pressure gradient; *TAPSE*, tricuspid annular plane systolic excursion; *RV-FAC*, right ventricular fractional area change; *LVEF*, left ventricular ejection fraction.

**Table E3:** Stratified analysis by annuloplasty ring type and severe-or-greater tricuspid regurgitation recurrence at 1 year

Stratum	2/3-leaflet, n/N (%)	4/5-leaflet, n/N (%)	OR (95% CI)	Wald *P*
Primary end point stratified by annuloplasty ring type				
Rigid Physio ring	11/30 (36.7)	13/23 (56.5)	2.25 (0.74-6.81)	.153
Flexible Tri-Ad band	7/37 (18.9)	3/10 (30.0)	1.84 (0.38-8.94)	.452

End point	2/3-leaflet, n/N (%)	4/5-leaflet, n/N (%)	*P* value
Severe-or-greater (≥3+) tricuspid regurgitation recurrence at 1 y			
Severe-or-greater TR recurrence at 1 y	0/68 (0.0)	1/33 (3.0)	.327

The primary end point was a Doppler-derived TRPG increase, defined as ΔTRPG ≥5 mm Hg from discharge to 1 year. Stratified estimates are directionally consistent across ring strata, but each stratum is small and the analysis is exploratory. Severe-or-greater TR recurrence was defined as grade ≥3+ on the institutional ordinal scale, corresponding to severe-or-greater regurgitation by American Society of Echocardiography/European Association of Cardiovascular Imaging criteria; the denominator is the number of patients with an evaluable 1-year TR grade. The single recurrence occurred in a patient with four-leaflet anatomy (duplicated anterior leaflet) whose TR grade trajectory was 3+ preoperatively, 1+ at discharge, and 3+ at 1 year. *OR*, Odds ratio; *TR*, tricuspid regurgitation; *TRPG*, tricuspid regurgitation pressure gradient.

**Table E4:** Complete baseline clinical and echocardiographic characteristics

Variable	Total (N = 117)	2/3-leaflet (n = 75)	4/5-leaflet (n = 42)	*P* value
Age, y	72.1 ± 9.6	70.4 ± 10.6	75.1 ± 6.6	.004
Female, n (%)	57 (48.7)	33 (44.0)	24 (57.1)	.184
Body mass index, kg/m²	22.1 ± 5.5	22.7 ± 6.4	21.2 ± 3.3	.105
Body surface area, m²	1.60 ± 0.21	1.63 ± 0.21	1.54 ± 0.18	.017
Sinus rhythm, n (%)	31 (26.5)	22 (29.3)	9 (21.4)	.391
Atrial fibrillation, n (%)	85 (72.6)	53 (70.7)	32 (76.2)	.666
NYHA class III or IV, n (%)	43 (36.8)	24 (32.0)	19 (45.2)	.167
Hypertension, n (%)	30 (25.6)	17 (22.7)	13 (31.0)	.380
Diabetes mellitus, n (%)	17 (14.5)	9 (12.0)	8 (19.0)	.412
Chronic kidney disease, n (%)	56 (47.9)	32 (42.7)	24 (57.1)	.177
Previous stroke, n (%)	11 (9.4)	8 (10.7)	3 (7.1)	.744
Chronic obstructive pulmonary disease, n (%)	4 (3.4)	3 (4.0)	1 (2.4)	1.000
Liver cirrhosis, n (%)	3 (2.6)	1 (1.3)	2 (4.8)	.292
Coronary artery disease, n (%)	18 (15.4)	11 (14.7)	7 (16.7)	.794
TR etiology, n (%)				.601
Primary	11 (9.4)	6 (8.0)	5 (11.9)	
Secondary (atrial- and ventricular-functional)	100 (85.5)	66 (88.0)	34 (81.0)	
CIED-related	6 (5.1)	3 (4.0)	3 (7.1)	
Severe-or-greater preoperative TR, n/N (%)	32/114 (28.1)	20/75 (26.7)	12/39 (30.8)	.665
EuroSCORE II, %	2.84 [1.88-5.24]	2.50 [1.58-4.73]	3.98 [1.99-5.50]	.037
STS-PROM, %	3.9 [2.2-7.1]	3.8 [2.0-7.4]	4.1 [3.4-6.9]	.345
TRI-SCORE, %	3 [2-8]	3 [2-8]	4 [2-8]	.339
Preoperative TRPG, mm Hg	35.0 ± 13.4	35.9 ± 13.6	33.5 ± 13.0	.338
Preoperative TAPSE, cm	1.92 ± 0.55	1.87 ± 0.55	2.01 ± 0.53	.215
Preoperative RV-FAC, %	38.0 ± 6.6	37.7 ± 6.2	38.6 ± 7.4	.492
Preoperative LVEF, %	58.9 ± 9.7	58.9 ± 10.4	58.9 ± 8.4	.968
Left atrial diameter, cm	5.0 ± 1.1	5.0 ± 1.0	5.0 ± 1.3	.789
Preoperative tricuspid annular diameter, mm	39.7 ± 6.1	39.5 ± 4.9	40.1 ± 7.6	.683
Preoperative TR vena contracta width, mm	9.9 ± 3.8	9.1 ± 2.5	11.0 ± 4.8	.066
Preoperative serum creatinine, mg/dL	1.26 ± 1.35	1.26 ± 1.37	1.28 ± 1.32	.945

Data are mean ± SD, median [interquartile range], or n (%). Continuous variables were compared by the Welch *t* test or the Mann-Whitney *U* test according to distribution; categorical variables by the Fisher exact test, including its extension for the 2×3 comparison of TR etiology. Interquartile ranges use the type-7 quantile definition with linear interpolation. Severe-or-greater TR denotes grade ≥3+ on the institutional ordinal scale, corresponding to severe-or-greater regurgitation by American Society of Echocardiography/European Association of Cardiovascular Imaging criteria. Variables with more than 5% missingness were EuroSCORE II (91 of 117 evaluable), preoperative TR vena contracta width (61 of 117), preoperative tricuspid annular diameter (70 of 117), and preoperative TAPSE (110 of 117); severe-or-greater preoperative TR was evaluable in 114 of 117. All other variables had ≤5% missingness. Comparisons are reported as 4/5-leaflet versus 2/3-leaflet morphology. *NYHA*, New York Heart Association; *TR*, tricuspid regurgitation; *CIED*, cardiac implantable electronic device; *EuroSCORE*, European System for Cardiac Operative Risk Evaluation; *STS-PROM*, Society of Thoracic Surgeons Predicted Risk of Mortality; *TRPG*, tricuspid regurgitation pressure gradient; *TAPSE*, tricuspid annular plane systolic excursion; *RV-FAC*, right ventricular fractional area change; *LVEF*, left ventricular ejection fraction.

## Appendix E1. Methods

### Data Sources

Clinical demographics, comorbidities, risk scores, and operative variables were drawn from the prospective surgical database. All echocardiographic measurements, serum creatinine, follow-up duration, and mortality were drawn from the prospectively maintained echocardiographic follow-up database, which served as the reference source after a structured data audit identified unit-entry errors in the surgical database echocardiographic fields. Two individual corrections were applied: 1 patient whose tricuspid annular diameter was recorded as 3.8 (cm entered in a millimeter field) was corrected to 38 mm, and 1 patient whose intraoperative leaflet count was recorded as indeterminate was classified as two-leaflet anatomy on the operating surgeon's definitive assessment.

### Distributional Assessment and Descriptive Statistics

Continuous variables were assessed for normality by the Shapiro-Wilk test together with inspection of skewness and quantile–quantile plots. Normally distributed variables are summarized as mean ± SD and compared by the Welch *t* test; non-normally distributed variables are summarized as median [interquartile range] and compared by the Mann-Whitney *U* test. Interquartile ranges were calculated using the type-7 quantile definition with linear interpolation, the default in R. Categorical variables are summarized as n (%) and compared by the Fisher exact test, including its extension for larger contingency tables where more than 2 categories were compared. Within-group change from discharge to 1 year was assessed by the Wilcoxon signed-rank test.

### Handling of Missing Data

All analyses are complete-case; no imputation was performed. Denominators are reported for every variable with missing observations. Of the 117 enrolled patients, 100 (85.5%) had paired discharge and 1-year tricuspid regurgitation pressure gradient (TRPG) measurements and constituted the primary analysis population. The 17 excluded patients comprised 11 in whom 1-year echocardiography was not performed, 4 in whom TRPG was not measurable because regurgitation was absent or trivial, and 2 in whom echocardiographic data were unavailable at both time points. Baseline characteristics of the analyzed and excluded patients are compared in Table E2. In the prespecified missingness sensitivity analysis, the 4 patients with nonmeasurable 1-year TRPG owing to absent or trivial regurgitation were reclassified as nonevents, yielding a denominator of 104.

### Multivariable Modeling

Covariates were prespecified a priori on the basis of clinical relevance and biologic plausibility rather than by univariable screening: leaflet morphology (the principal exposure); age (which differed at baseline and is a recognized determinant of right-heart trajectory); rigid Physio ring use (institutional preferential allocation to patients with more severe baseline regurgitation, with potential biomechanical relevance); and concomitant spiral suspension (which targets the posterolateral substrate disproportionately represented in the multi-leaflet phenotype). Logistic regression was constructed sequentially as a univariable model, an age-adjusted model, and a full model. Odds ratios and 95% CIs are Wald-based, and the analysis is interpreted as explanatory rather than predictive. The full model showed acceptable goodness-of-fit (Hosmer-Lemeshow *P* = .85 based on deciles of predicted probability) and modest discrimination (c-statistic 0.68). Because the events-per-variable ratio was approximately 8.5 (34 events across 4 covariates), Firth's penalized likelihood was applied as a sensitivity analysis to address small-sample bias; the Firth-adjusted estimate for 4/5-leaflet morphology remained directionally consistent.

### Sensitivity Analyses

Three prespecified sensitivity analyses examined the robustness of the 5-mm Hg operational threshold: alternative cutoffs of 3 mm Hg and 10 mm Hg, a comparison of continuous ΔTRPG, and the missingness reclassification described previously. A stratified analysis by annuloplasty ring type was performed to examine whether the association between leaflet morphology and the primary end point differed by prosthesis. The primary end point constituted the single prespecified primary comparison; all other analyses are secondary or exploratory and no adjustment for multiplicity was applied. We performed analyses using R, version 4.3, or greater.
